# Should we still be performing macular laser for non-centre involving diabetic macular oedema? Results from a UK centre

**DOI:** 10.1038/s41433-021-01789-3

**Published:** 2021-10-21

**Authors:** Peter H. Scanlon, Charlotte F. E. Norridge, Paul H. J. Donachie, Quresh Mohamed

**Affiliations:** 1 Gloucestershire Retinal Research Group, Gloucestershire Hospitals NHS Foundation Trust, Cheltenham General Hospital, Cheltenham, UK; 2 Nuffield Department of Clinical Neuroscience, University of Oxford, Oxford, UK; 3 University of Gloucestershire, Cheltenham, UK

**Keywords:** Outcomes research, Surgery

## Background

The Early Treatment Diabetic Retinopathy Study [1] (ETDRS) showed that focal photocoagulation of “clinically significant” diabetic macular oedema (CSMO) substantially reduced the risk of visual loss. Vascular Endothelial Growth Factor (VEGF) inhibitor drugs have subsequently been shown to have better results for centre involving diabetic macular oedema [2].

## Methods

**Fig. 1:**
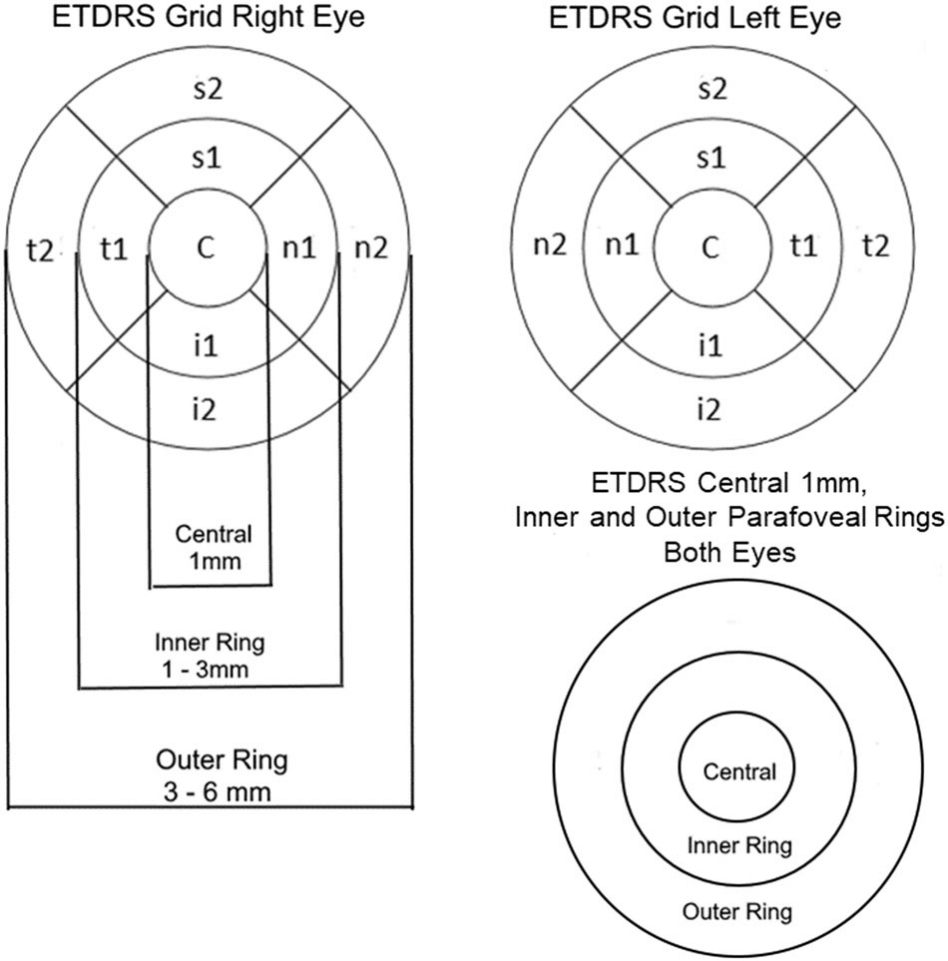
Early treatment diabetic retinopathy study (ETDRS) grid areas. ETDRS grid right eye, ETDRS grid left eye, ETDRS central 1 mm, inner and outer parafoveal rings both eyes.

A retrospective analysis of first macular laser treatment was conducted between 01/01/2010 and 31/12/2019 and OCT measurements from the same machine, within 3 months before and 1–12 months after treatment in the Gloucestershire Eye Unit. Eyes were excluded if their central subfield retinal thickness (CRT) was ≥ 400 microns, they had ocular co-pathologies or surgery in the previous 6 months. ETDRS grid areas described by Soliman [3] were used to assess the results of laser treatment (Fig. 1).

## Results

Eligible for analysis were 316 eyes from 275 patients with 175 male, 246 T2DM, and median age 63.1 years. A total of 8 consultants treated 198 (62.7%) eyes, 5 specialty doctors treated 101 (32.0%) eyes and 11 trainees treated 17 (5.4%) eyes with 2 consultants treating >50 eyes. The laser machine used was a PASCAL green laser (Topcon UK) for 271 (85.8%) eyes. The median number of burns administered to an eye was 24 (IQR; 15–34). Burn durations of 0.02 or 0.05 s were used for 69.3% of eyes. For 311 (98.4%) eyes the beam diameter was 100 microns.

A total of 253 eyes had Zeiss Cirrus OCT measurements (Table 1), mean baseline CRT 303.6 microns, mean follow-up CRT 305.1 microns and mean difference +1.5 microns (95% CI: −4.8 to +7.7). In only two macular regions did <50% of eyes have a reduction in thickness. A total of 63 eyes had Heidelberg OCT measurements, mean baseline CRT 302.4 microns, mean follow-up CRT 309.4 microns and mean difference +7.0 microns (95% CI: −4.7 to +18.8). In all macular regions >50% of eyes had a reduction in thickness.

**Table 1:** Macular Thickness estimates for 253 eyes assessed using a Zeiss Cirrus OCT machine and 63 eyes assessed using the Heidelberg OCT machine.

Macular thickness (µm)	Baseline mean (microns)	Follow-up mean (microns)	Mean difference^a^ (microns)	95% CI^b^ (microns)	Reduction *n* (%)	No change *n* (%)	Increase *n* (%)
Zeiss Cirrus (*N* = 253 unless^c^)							
Macular region							
Temporal t1	352.0	347.4	−4.6	−10.028 to 0.732	147 (58.1)	6 (2.4)	100 (39.5)
Superior s1	353.6	347.2	−6.4	−10.542 to −2.296	141 (55.7)	7 (2.8)	105 (41.5)
Inferior i1	337.8	337.5	−0.3	−4.358 to 3.765	126 (49.8)	13 (5.1)	114 (45.1)
Nasal n1	338.6	337.8	−0.8	−5.262 to 3.570	138 (54.5)	12 (4.7)	103 (40.7)
Temporal t2^c^	311.2	304.3	−6.8	−11.616 to −2.019	138 (57.3)	5 (2.1)	98 (40.7)
Superior s2^c^	310.8	306.9	−3.6	−7.281 to −0.007	142 (56.8)	2 (0.8)	106 (42.4)
Inferior i2^c^	296.0	294.6	−1.7	−5.756 to 2.346	114 (46.7)	17 (7.0)	113 (46.3)
Nasal n2^c^	310.9	309.9	−1.0	−4.270 to 2.343	127 (51.6)	14 (5.7)	105 (42.7)
ETDRS circle							
ETDRS 1 mm (Central C)	303.6	305.1	1.5	−4.750 to 7.659	130 (51.4)	7 (2.8)	116 (45.8)
Inner parafoveal ring (1–3 mm)	1,382.0	1,369.8	−12.2	−24.399 to −0.020	149 (58.9)	1 (0.4)	103 (40.7)
Outer parafoveal ring (3−6 mm)^c^	1,226.1	1,212.7	−12.6	−22.288 to −2.900	134 (58.5)	0 (0.0)	95 (41.5)
Mean Cube average thickness^c^	306.4	303.5	−2.9	−5.626 to −0.175	144 (57.4)	5 (2.0)	102 (40.6)
Mean total macular volume	11.0	10.9	−0.1	−0.211 to −0.022	142 (56.1)	15 (5.9)	96 (37.9)
Heidelberg (*N* = 63 unless^c^)							
Macular region							
Temporal t1	356.2	359.4	3.2	−5.063 to 11.444	33 (52.4)	1 (1.6)	29 (46.0)
Superior s1	368.2	366.9	−1.3	−7.954 to 5.351	37 (58.7)	2 (3.2)	24 (38.1)
Inferior i1	354.9	357.8	2.9	−5.912 to 11.721	36 (57.1)	0 (0.0)	27 (42.9)
Nasal n1	357.2	355.8	−1.4	−8.302 to 5.477	37 (58.7)	6 (9.5)	20 (31.7)
Temporal t2	328.6	326.3	−2.3	−9.020 to 4.416	36 (57.1)	2 (3.2)	25 (39.7)
Superior s2	330.2	333.7	3.5	−9.750 to 16.844	36 (57.1)	0 (0.0)	27 (42.9)
Inferior i2^c^	322.6	318.3	−4.6	−11.981 to 2.833	37 (60.7)	2 (3.3)	22 (36.1)
Nasal n2	333.4	332.1	−1.3	−6.239 to 3.572	33 (52.4)	5 (7.9)	25 (39.7)
ETDRS circle							
ETDRS 1 mm (Central C)	302.4	309.4	7.0	−4.733 to 18.764	29 (46.0)	0 (0.0)	34 (54.0)
Inner parafoveal ring (1–3 mm)	1,436.6	1,439.9	3.4	−22.173 to 28.935	36 (57.1)	0 (0.0)	27 (42.9)
Outer parafoveal ring (3–6 mm)^c^	1,312.8	1,308.7	−4.7	−27.937 to 18.575	40 (63.6)	0 (0.0)	21 (34.4)
Mean total macular volume	9.5	9.4	−0.1	−0.215 to 0.080	18 (28.6)	19 (30.2)	26 (41.3)

^a^Negative values indicate reduction in thickness and positive values increase in thickness.

^b^95% Confidence Interval for the mean difference in measurements between baseline and follow up.

^c^Due to OCT machines not being able to measure all regions some eyes have missing values. Zeiss Cirrus assessed eyes; for the temporal t2 region (*n* = 248 at baseline, 244 at follow up and 241 for difference). For the superior s2 region (*n* = 252 at baseline, 251 at follow up and 250 for difference). For the inferior i2 region (*n* = 247 at baseline, 250 at follow up and 244 for difference). For the nasal n2 region (*n* = 249 at baseline, 250 at follow up and 246 for difference). For the outer parafoveal ring (*n* = 239 at baseline, 241 at follow up and 229 for difference). For the mean cube average (*n* = 252 at baseline, 251 at follow up and 251 for difference). Heidelberg assessed eyes; for the inferior i2 region (*n* = 62 at baseline, 61 at follow up and 61 for difference). For the outer parafoveal ring (*n* = 62 at baseline, 61 at follow up and 61 for difference).

After VEGF inhibitor injections became available in 2013, only 3 (5.4%) eyes with Heidelberg and 10 (10.5%) eyes with Zeiss OCT measurements received injections within 1 year of laser.

The median baseline and follow up Visual Acuity (VA) were both 0.20 LogMAR.

## Discussion

The Diabetic Retinopathy Clinical Research Network (DRCRN) [4] compared two laser techniques in 2007 that had OCT data available on 213 eyes at 12 months post laser. They found, in both treatment groups, a reduction of central retinal thickening, weighted inner zone thickening, and retinal volume with no significant change in visual acuity outcomes.

As the NICE guidelines [5] in England do not recommend treatment until the central subfield retinal thickness (CRT) is ≥400 microns, macular laser treatment was assessed for predominantly non-centre involving diabetic macular oedema.

Macular laser treatment can be effective in reducing retinal thickening in the inner and outer parafoveal zones and in different macular regions with stable VA. Only 5.1% of eyes went on to require injections with VEGF inhibitors within 12 months of the initial laser. This study provides evidence that there is still a place for macular laser treatment in non-centre involving diabetic macular oedema.
